# The Protective Effect of Aspirin against Myocardial Hypertrophy in Rats

**DOI:** 10.1155/2021/2043415

**Published:** 2021-04-20

**Authors:** Xiaolong Wu, Minghui Wei, Haifeng Zhang, Xiaomei Fan, Xiaochen Ma, Jiaming Liu, Mingming Xue

**Affiliations:** ^1^Department of Physiology, Inner Mongolia Medical University, Hohhot, Inner Mongolia 010110, China; ^2^Department of Laboratory, Baotou Central Hospital, Baotou city, Inner Mongolia 014040, China; ^3^School of Basic Sciences, Inner Mongolia Medical University, Hohhot, Inner Mongolia 010110, China

## Abstract

The protective effect of aspirin against myocardial hypertrophy (MH) was studied. Model rats of pressure overload MH were prepared by abdominal aortic coarctation. Rats were randomly divided into the sham group (*n* = 9), MH model group (*n* = 9), and MH+aspirin group (*n* = 9), which was, respectively, divided into the 4-week group and 8-week group according to the time of intragastric administration. Arterial blood pressure and left ventricular mass index (LVMI) were measured. Changes in myocardial tissue structure were observed by HE staining, Masson staining, and reticular fiber staining. Cardiomyocyte apoptosis was detected by TUNEL assay. The levels of TNF-*α*, IL-10, TXA2, and PGI2 in myocardium and plasma were detected by ELISA. The arterial blood pressure in the MH model group was significantly higher than that in the 4- and 8-week sham groups, but that in the MH+aspirin group was significantly lower than that in the MH model group. At 4 and 8 weeks, the LVWI in the MH model group was significantly higher than that in the sham group, but it was significantly reduced after aspirin treatment. The myocardial cell hypertrophy was obvious, collagen fibers were proliferated, and reticular fibers were reduced in the 4- and 8-week MH model groups. Compared with the MH model groups, myocardial cells in the MH+aspirin groups were significantly reduced, the collagen fiber content was significantly reduced, and the reticular fiber content was increased. The apoptotic cardiomyocytes in the 4- and 8-week MH model groups were obviously increased. The apoptosis of myocardial cells in the MH+aspirin groups was obviously decreased. The TNF-*α* levels in the myocardial tissue of the 4- and 8-week MH model groups were significantly increased, while those of the MH+aspirin groups were significantly decreased. There was no significant change in the IL-10 level or PGI2 level at 4 weeks. At 8 weeks, the PGI2 level was significantly decreased in the MH model group while significantly increased in the MH+aspirin group. The TXA2 levels were significantly increased in the 4- and 8-week MH model groups and those in the 4- and 8-week MH+aspirin groups were significantly lower. Aspirin has an anti-inflammatory effect, can effectively reduce the expression of inflammatory factors, inhibit myocardial apoptosis, and has a certain protective effect against MH.

## 1. Introduction

Myocardial hypertrophy (MH), an adaptive response to myocardial overload, is one of the independent risk factors for various cardiovascular diseases [1]. It is characterized by the proliferation of myocardial connective tissue and the increase of cell volume. The persistent MH is often an early manifestation of heart failure [2]. The pathogenesis of MH is unclear. Studies have shown that it may be related to inflammation, neurohumoral factors, and endocrine factors [3, 4].

Studies have shown that a large number of inflammatory factors participate in the formation of MH (including ventricular remodeling, fibrosis, and apoptosis) [5], including tumor necrosis factor (TNF-ɑ) and interleukin 6 (IL-6) and other anti-inflammatory factors such as interleukin 10 (IL-10) [6]. It is currently believed that TNF-*α* has effects on many aspects of MH. TNF-*α* can induce cardiomyocytes to undergo apoptosis, cause cardiomyocytes to hypertrophy, and can bidirectionally regulate the production and degradation of collagen fibers. IL-10 is a major anti-inflammatory factor that inhibits the synthesis and biological effects of many inflammatory factors [7]. The regulation between inflammatory factors is very complex. Proinflammatory and anti-inflammatory factors can both promote each other and antagonize each other. They maintain a balance to maintain the body's homeostasis [8].

Cyclooxygenase (COX) has two isoforms COX-1 and COX-2. COX-1 plays a role in homeostasis and is constitutively expressed. However, COX-2 expression can be induced by inflammation [9]. Thus, COX-2 is an important proinflammatory cell factor and is associated with proinflammatory factors such as TNF-*α* and IL-6 and anti-inflammatory factors such as IL-10 [9]. Aspirin is a nonselective COX inhibitor that blocks COX and reduces the production of Prostaglandin (PG) and ThromboxaneA2 (TXA2) [10, 11]. Aspirin inhibits the metabolism of arachidonic acid by inhibiting the activity of COX and finally inhibits the synthesis of TXA2 and PG to achieve anti-inflammatory effects [12, 13]. Aspirin is used for the prevention of many types of diseases, including cardiovascular disease [14].

Apoptosis is involved in many cardiovascular diseases such as hypertension, cardiac hypertrophy, and atherosclerosis [15]. During the development of MH, cardiomyocyte apoptosis plays an important role.

In this study, we aim to investigate the protective effect of aspirin against MH. The changes of inflammatory factors during the development of MH were analyzed. The effects of aspirin on the left ventricular structure of MH rats and the relationship between aspirin and inflammatory factors TNF-*α*, IL-10, PGI2, and TXA2 were investigated.

## 2. Materials and Methods

### 2.1. Animals

Wister male rats weighing about 230 g (*n* = 54; 10-week-old) were obtained from the Animal Experimental Center of Inner Mongolia University. All animal experiments were conducted according to the ethical guidelines of Inner Mongolia University (approval number: SCXK(Mongolia)2016-0001).

### 2.2. Model Establishment and Grouping

MH was established by abdominal aortic coarctation [16]. Briefly, rats were anesthetized with 2% pentobarbital sodium (i.p.; 40 mg/kg). A midline incision was made 2 to 3 cm below the xiphoid of rats. After exposing the abdominal cavity, the peritoneum, and the left kidney, the abdominal aorta was isolated. The ligation was performed at 0.5 to 1.5 cm of the abdominal aorta where an obvious pulsation could be felt. The pentobarbital sodium was also used for postoperative analgesia. The surgery success rate was over 80% with 1-2 deaths in each group. After model establishment, the rats were randomly divided into the MH model group (*n* = 18) and MH+aspirin group (*n* = 18). In the sham group (*n* = 18), the same procedures were performed but without ligation. Rats in the MH model and the sham group were intragastrically administered with normal saline on the next day after operation. Rats in the MH+aspirin group were intragastrically administered with 10 mg/mL aspirin solution (200 mg/kg/day). Aspirin was administered chronically for 4 or 8 weeks. The above three groups were randomly divided into the 4-week group and 8-week group according to the time of intragastric administration.

### 2.3. Noninvasive Arterial Blood Pressure Measurement

The systolic blood pressure (SBP) and diastolic blood pressure (DBP) of each group were measured by automatic noninvasive blood pressure measuring instrument BP-300A (CDTME, Chengdu, China) at 4 and 8 weeks after operation. The arterial blood pressure values were measured 3 times and averaged.

### 2.4. Collection of Cardiac Blood and Myocardial Tissue Specimens

At the end of the experiment, the rats were anesthetized with 2% sodium pentobarbital. The cardiac blood was collected from the heart, and the serum was isolated. After that, the rats were sacrificed by decapitation. Then, the heart was dissected, blotted by the filter paper, and weighed. The left ventricle was weighed, and the left ventricular mass index (LVMI) was calculated. The myocardial tissue was taken along the long axis of the left ventricle. Some of the myocardial tissue was fixed with 10% neutral formalin and made into paraffin sections. Some of the myocardial tissue was stored in liquid nitrogen, which was used in ELISA to detect the content of inflammatory factors in myocardial tissue.

### 2.5. Morphological Changes of Left Ventricular Structure

HE staining, Masson staining, and modified Gomori reticular fiber staining were used to observe left ventricular morphological structure and fiber changes. The protocols were performed according to routine procedure. Briefly, for HE staining, the sections were dewaxed, stained with hematoxylin-eosin, dehydrated and transparentized, and mounted. For Masson's trichrome staining, the sections were dewaxed, stained with hematoxylin followed by Masson staining, dehydrated and transparentized, and mounted. For the modified Gomori reticular fiber staining, the sections were dewaxed, stained with Gomori ammonia silver, dehydrated and transparentized, and mounted. Finally, the morphological changes were observed under light microscope.

To calculate the average cardiomyocytes diameter, 10 fields of each specimen were randomly selected under a 400x microscope, and 10 cells were selected from each field. The area of each cell was measured with Image-Pro Plus, and the average diameter was calculated after averaging.

To calculate the collagen volume fraction (CVF), the Leica Application Suite-LAS system was used to process the image under 200x magnification. One slice was selected for each specimen, and 6 regions without coronary vessels and 6 regions with coronary vessels were selected for each slice. CVF was calculated as the average percentage of collagen fibers. The CVF of the coronary arterial visual field was defined as the percentage of total left ventricular myocardial collagen volume fraction (CVF-T). The visual field collagen volume fraction percentage was abbreviated as (CVF-NV).

One section was selected for each specimen, and six fields of view were selected for each section. The volume percentage of the reticular fibers in each field of vision was calculated and defined as volume percentage (RVF-T).

### 2.6. TUNEL

Cardiomyocyte apoptosis was detected by TUNEL assay according to the manufacturer's instructions (Promega, Madison, Wisconsin, USA). The nuclei were stained with DAPI. The green fluorescence, which indicates the apoptosis, was observed at 520 ± 20 nm. The blue fluorescence, which indicates the nuclei, was observed at 460 nm. The apoptosis rate was calculated as number of apoptotic cells (TUNEL positive)/total number of cells (DAPI positive) × 100%.

### 2.7. ELISA

The cardiac blood was collected from the heart, and the serum was isolated. The myocardial tissue was homogenized, and the supernatant was collected after centrifugation at 5000 r/min for 15 min. The levels of IL-10, PGI_2_, TXA_2_, and TNF-*α* in the serum and myocardial tissue supernatant were determined with a Rat ELISA test kit according to the manufacturer's instructions (Biowinner, Beijing, China). HRP-labeled goat anti-rabbit IgG (1 : 5) were used as the secondary antibody. The absorbance value at 450 nm wavelength was read with a microplate reader (Multiskan MK-3, Finland).

### 2.8. Statistical Analysis

The data was analyzed by SPSS 13.0 (IBM, Armonk, NY, USA). Data was described as mean ± standard deviation. The *t*-test and SNK-*q* test were used to compare the differences between different groups at the same time point. *P* value < 0.05 was considered statistically significant.

## 3. Results

### 3.1. MH Model Is Established in Rats

To test if the rat model is successfully established, blood pressure was measured at different time points. Compared with the sham group, the blood pressure (SBP and DBP) (Table 1) was increased, and the difference was statistically significant (*P* < 0.05). LVMI was also calculated. As shown in Table 2, LVMI in the MH model group was significantly increased than that in sham group (*P* < 0.05). However, there was a significant decrease in LVMI of the MH+aspirin group than that of the MH model group (*P* < 0.05). These showed that the model rats with MH were successfully established.

### 3.2. Changes of Ventricular Structure in Model Rats with MH

**Figure 1:**
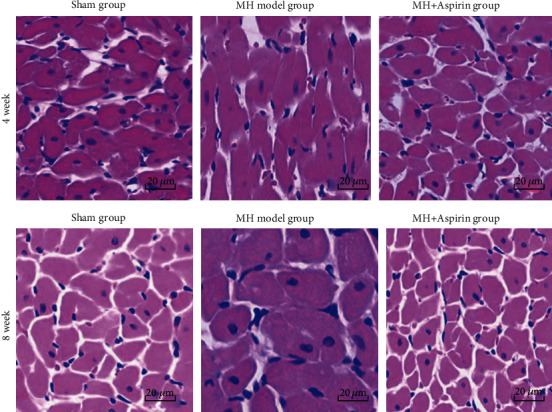
Pathological morphology of myocardial tissue in each group (*n* = 9). Myocardial tissues were collected from the sham group, MH model group, and MH+aspirin group at 4 weeks and 8 weeks after model establishment. HE staining was performed to observe the pathological changes. Representative images were shown. Magnification: 400x.

After HE staining, the myocardial cells in the 4-week MH model group were disordered, hypertrophied, and irregular in morphology (Figure 1). The MH was more obvious, and there were inflammatory cells in the 8-week MH model group (Figure 1). Compared with the MH model group, the average cardiomyocyte diameter in the 4-week and 8-week MH+aspirin groups was significantly reduced (Table 3).

**Figure 2:**
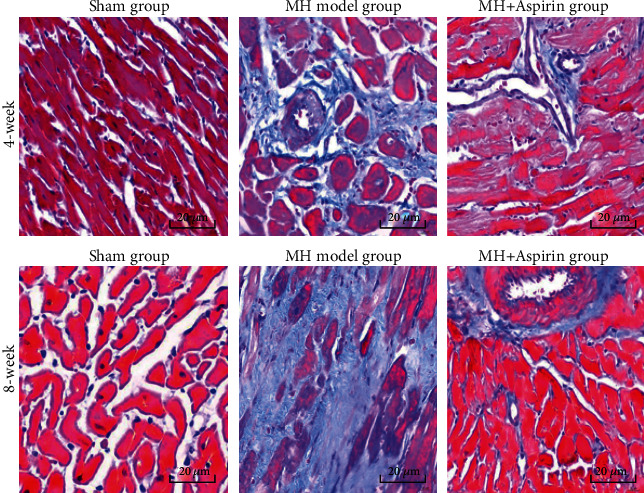
Collagen fiber analysis of myocardial tissue in each group (*n* = 9). Myocardial tissues were collected from the sham group, MH model group, and MH+aspirin group at 4 weeks and 8 weeks after model establishment. Masson staining was performed to observe the collagen fibers. Representative images were shown. Magnification: 400x.

After Masson staining, the collagen fibers showed focal hyperplasia (mainly perivascular hyperplasia) in the 4-week MH model group (Figure 2). The myocardial injury was more serious, and the collagen fibrosis was more obvious in the 8-week MH model group (Figure 2). The collagen fibers in the 4- and 8-week MH+aspirin groups were more proliferative than the sham group, but not as obvious as the MH model group (Figure 2). CVF-T and CVF-NV significantly increased in the 4-week and 8-week MH model groups compared with the sham group (Table 4), and the CVF-NV of the 4-week and 8-week MH+aspirin groups were significantly lower than the MH model group (Table 4). CVF-T of the MH+aspirin group decreased significantly compared with the MH model group (Table 4).

**Figure 3:**
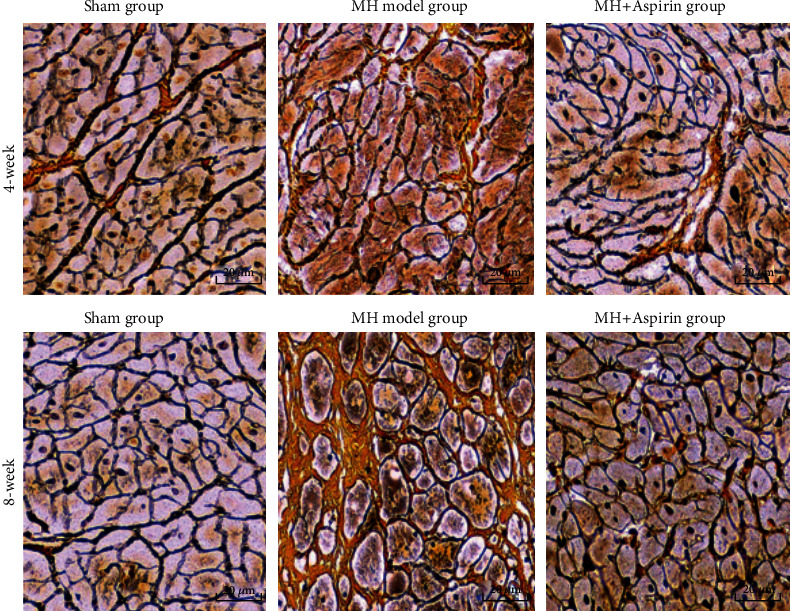
Pathological morphology of myocardial tissue in each group (*n* = 9). Myocardial tissues were collected from the sham group, MH model group, and MH+aspirin group at 4 weeks and 8 weeks after model establishment. Modified Gomori reticular fiber staining was performed to observe the fibers. Representative images were shown. Collagen fibers were yellow, reticular fibers were black, and cardiomyocytes were pale yellow or colorless. Magnification: 400x.

Gomori reticular fiber staining showed that the reticular fibers in the 4- and 8-week MH model groups were obviously reduced (Figure 3). The 4-week and 8-week MH+aspirin group had obviously increased reticular fibers compared with the MH model group (Figure 3). The RVF-T in the 4-week and 8-week MH model groups were significantly less than the sham group (Table 5), and the RVF-T in the 4-week and 8-week MH+aspirin groups increased significantly compared with the MH model group (Table 5). These results demonstrate that there is cardiac hypertrophy, collagen fibrosis, and reticular fiber reduction in the early stage of MH.

### 3.3. Cardiomyocyte Apoptosis in Rats

**Figure 4:**
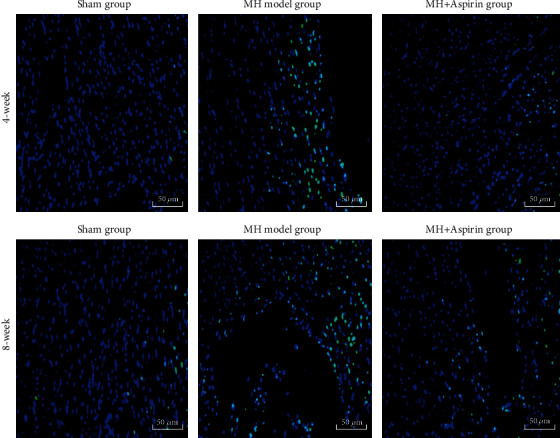
Cardiomyocyte apoptosis of rats in each group (*n* = 9). Myocardial tissues were collected from the sham group, MH model group, and MH+aspirin group at 4 weeks and 8 weeks after model establishment. Cardiomyocyte apoptosis was detected by TUNEL assay. Representative images were shown. The apoptosis is indicated as green fluorescence, and the nuclei are shown as blue fluorescence. Magnification: 400x.

TUNEL was performed to analyze apoptosis. As shown in Figure 4 and Table 6, compared with the sham group, the apoptotic cardiomyocytes in the 4- and 8-week MH model groups were obviously increased. Compared with the MH model group, the apoptosis of myocardial cells in the MH+aspirin groups was obviously decreased.

### 3.4. Effect of Aspirin on Arterial Blood Pressure in Model Rats with MH

The effect of aspirin on arterial blood pressure in rats with MH was analyzed. The SBP and DBP were measured at 4 weeks and 8 weeks. Compared with the sham group at the same time point, the SBP and DBP in the MH model group were increased, and the differences were statistically significant (Table 1). Compared with the MH model group, the SBP and DBP in the MH+aspirin group were decreased, and the difference was statistically significant (Table 1). These results indicate arterial blood pressure is significantly increased in rats with MH, and this increase was reduced by aspirin.

### 3.5. Effect of Aspirin on Inflammatory Factors in Model Rats with MH

Inflammatory factors were detected by ELISA. The TNF-*α* level in the serum had no significant difference between the 4 and 8 weeks of the rats in each group (Table 7). The TNF-*α* levels in the myocardial tissue of the 4- and 8-week MH model groups were significantly higher than those in the sham groups (*P* < 0.05). The TNF-*α* levels in myocardial tissue of the 4- and 8-week MH+aspirin groups were significantly lower than those in the MH model groups (*P* < 0.05). There was no significant change in the IL-10 level in the plasma and myocardial tissue between the 4 and 8 weeks of the rats in each group (Table 8). There was no significant change in the PGI_2_ level in the plasma and myocardial tissue among three groups at 4 weeks (Table 9). The PGI_2_ level in the plasma and myocardial tissue was significantly decreased in the MH model group at 8 weeks as compared with that in the sham group (*P* < 0.05). At week 8, the MH+aspirin group had significantly higher level of PGI_2_ than the MH model group (*P* < 0.05). The TXA_2_ levels in the plasma and myocardial tissues of the 4- and 8-week MH model groups were increased as compared with those in the sham group, and the difference was statistically significant (Table 10) (*P* < 0.05). Compared with the MH model group, the TXA_2_ levels in the myocardial tissue and plasma of the MH+aspirin group were significantly lower (*P* < 0.05). These results suggest that TNF-*α* and TXA2 levels are significantly increased in rats with MH, while the PGI2 level is significantly decreased. Aspirin can significantly reduce the levels of TNF-*α* and TXA2.

## 4. Discussion

MH is one of the independent risk factors in cardiovascular diseases. Inflammatory factors play an important role in the development of MH and are involved in ventricular remodeling [17]. Changes in blood pressure, LVMI, and pathological morphology are the primary indicators of MH [18]. In this study, we established an MH model according to previous description [19]. The blood pressure and LVMI were observed at 4 weeks and 8 weeks after model establishment. We found that the blood pressure and LVMI in the MH model group were significantly higher than those in the sham group, indicating that the MH model is successfully established.

Aspirin can reduce cardiac interstitial fibrosis by inhibiting Erk1/2-Serpine2 and P-Akt signaling pathways [20]. In humans, there is evidence that aspirin may impair reverse myocardial remodeling in heart failure patients treated with beta blockers [21]. Vascular endothelial injury can promote the release of many inflammatory factors, which can further stimulate collagen fibrosis and lead to myocardial fibrosis [22–26]. Meanwhile, they can activate thrombospondin-1 on platelet [27]. TGF-*β* can promote the expression of extracellular matrix and inhibit the degradation of collagen fibers and participate in the occurrence of myocardial fibrosis [28]. Liang et al. [29] showed that specific COX-2 inhibitors significantly reduced the collagen fraction of MH rats and improved myocardial collagen remodeling and cardiac function. In this study, changes in myocardial pathology were observed by HE staining, Masson staining, and reticular fiber staining. At 4 and 8 weeks, the MH model groups showed disordered myocardial cell arrangement, hypertrophy, focal hyperplasia of collagen fibers, and marked reduction of reticular fibers. However, myocardial cells and fibers in the MH+aspirin groups did not change obviously. The results indicate that aspirin can significantly inhibit collagen fibrosis and cardiomyocyte hypertrophy, thereby inhibiting ventricular fibrosis. This is consistent with the study of Liang et al. [29].

Okumura et al. found that COX inhibitors lowered blood pressure in a kidney-clamp hypertension model [30]. Our study showed that SBP and DBP were significantly higher in the MH model groups whereas they were significantly reduced in the MH+aspirin groups, which is consistent with the results of Okumura et al. [30]. Previous studies [31, 32] have shown that the imbalance between PGI_2_ and TXA_2_ is involved in the occurrence and development of hypertension, which is the cause of the continuous development of blood pressure [33]. PGI_2_ is one of the main metabolites of arachidonic acid. It has the functions of relaxing blood vessels, lowering blood pressure, and increasing blood flow of organs [34]. PGI_2_ can significantly reduce the pre- and postcirculation load and lower blood pressure, while TXA2 has the function of contracting blood vessels and raising blood pressure [35]. These two have antagonistic effects. Aspirin blocks the metabolism of arachidonic acid by inhibiting COX-1 [36] and significantly reduces the production of TXA2 without affecting the production of PGI2 [37], thereby lowering blood pressure. In this study, we found that at 8 weeks after model establishment, the PGI_2_ level in the MH model group was significantly decreased as compared with that in the sham group. The PGI_2_ level in the MH+aspirin group was significantly increased compared with that in the MH model group. This may be caused by the differences of different samples. Further studies are needed to clarify this. At 4 weeks and 8 weeks after model establishment, the TXA_2_ level in the plasma and myocardial tissue was significantly increased the MH model groups while that was significantly decreased in the MH+aspirin group. This may partly explain the decrease of blood pressure in MH rats intervened with aspirin.

The imbalance between proinflammatory and anti-inflammatory factors is one of important factors for the development of MH [38]. For example, the expression of inflammatory factors of TNF-*α* and IL-6 in the myocardial tissue of rats with MH was significantly increased [39]. Niu and Zhao [40] found that the expression of proinflammatory factors in the myocardial tissue was proportional to the level of collagen fibers in the myocardium, indicating that proinflammatory factors can promote the proliferation of collagen fibers in the myocardial tissue, leading to interstitial remodeling. Wang et al. [41] found that TNF-*α* induced MH in neonatal rats through the PI3K-IP3R-Ca^2+^ pathway. In this study, the TNF-*α* level was significantly increased in the MH model group whereas decreased in the MH+aspirin group. There was no significant change in IL-10 levels in the plasma and myocardial tissue. This result indicates that there may be imbalance of proinflammatory and anti-inflammatory factors in MH rats and aspirin administration may reverse this imbalance.

Furthermore, we found that the myocardial cell apoptosis rate in the MH model group increased significantly at 4 and 8 weeks after model establishment, but the MH+aspirin group significantly decreased the apoptosis rate compared with the MH model group. According to the results, it can be speculated that myocardial apoptosis seriously affects the development of MH. In the early stage of MH, cardiomyocytes undergo compensatory cell hypertrophy under the stimulation of pressure overload. Due to the stimulation of hypertrophic signals and in order to maintain myocardial structure and function, the myocardium initiates suicide mode and induces myocardial cell apoptosis; however, as time goes by, the stress overload stimulus persists, which may cause MH to decompensate, thereby increasing the myocardial cell apoptosis rate. In this process, aspirin can reduce the rate of myocardial apoptosis, delay the development of MH, and improve ventricular function [42]. On the other hand, aspirin could reduce TNF-*α* and IL-6 levels in patients with heart failure [43]. Studies have found that, in addition to the Caspase pathway, TNF-*α* can also induce cardiomyocyte apoptosis through the ceramide pathway [44, 45]. Thus, TNF-*α* produced by the myocardial tissue may ultimately affect cardiac function by inducing myocardial apoptosis. In this study, TNF-*α* in the myocardial tissue of the MH model group was significantly increased at 4 weeks. Under chronic pressure overload, the rate of TNF-*α*-induced cardiomyocyte apoptosis increased significantly at 8 weeks, confirming this statement. Moreover, the results of this study showed that aspirin significantly reduced the content of TNF-*α* produced by the myocardial tissue, which is consistent with a previous study [43]. Therefore, it can be speculated that aspirin may reduce the rate of cardiomyocyte apoptosis by reducing the content of TNF-*α* in the myocardial tissue, thereby alleviating MH and improving ventricular function.

This study has some limitations. First, the sample size was relatively small. Second, the mechanism underlying the protective effects of aspirin on MH was not investigated. Third, the long-term effects of aspirin were not evaluated. Further studies are warranted.

## 5. Conclusions

In conclusion, our findings demonstrate that aspirin had anti-inflammatory effects, effectively reduced the expression of inflammatory factors, inhibited myocardial apoptosis, and had a certain protective effect on the prevention of MH. This study may provide experimental evidence for the clinical treatment of MH.

## Figures and Tables

**Table 1:** Measurement of arterial blood pressure in each group (mean ± SD, *n* = 9 (mmHg)).

Groups	SBP		DBP	
	4-week	8-week	4-week	8-week
Sham	108.6 ± 5.45	110.4 ± 8.67	90.7 ± 4.17	85.6 ± 7.97
MH model	122.2 ± 13.07^∗^	128.9 ± 13.27^∗^	99.1 ± 6.28^∗^	108.1 ± 9.37^∗^
MH+aspirin	110.2 ± 6.15^△^	108.9 ± 6.97^△^	91.4 ± 6.51^△^	90.9 ± 5.32^△^

Note: ^∗^*P* < 0.05 compared with the sham group at the same time point. ^△^*P* < 0.05 compared with the MH model group at the same time point.

**Table 2:** Measurement of left ventricular weight, body weight, and LVMI in each group (mean ± SD, *n* = 9).

Groups	Left ventricular weight (g)		Body weight (g)		Left ventricular weight/body weight ×10^3^ (LVMI)	
	4-week	8-week	4-week	8-week	4-week	8-week
Sham	0.62 ± 0.08	0.71 ± 0.05	293.3 ± 23.50	344.8 ± 29.56	1.9 ± 0.28	2.2 ± 0.23
MH model	0.74 ± 0.10^∗^	0.83 ± 0.11^∗^	275.2 ± 16.06	296.2 ± 17.75^∗^	2.4 ± 0.21^∗^	2.9 ± 0.27^∗^
MH+aspirin	0.65 ± 0.17^△^	0.72 ± 0.17^△^	284.7 ± 11.17	322.8 ± 11.52	2.0 ± 0.13^△^	2.4 ± 0.11^△^

Note: LVMI: left ventricular mass index; ^∗^*P* < 0.05 compared with the sham group at the same time point. ^△^*P* < 0.05 compared with the MH model group at the same time point.

**Table 3:** Measurement of the average diameter of the myocardial cell in each group (mean ± SD, *n* = 9 (*μ*m)).

Groups	Average diameter of myocardial cell	
	4-week	8-week
Sham	9.02 ± 0.25	9.51 ± 0.33
MH model	9.89 ± 0.46^∗^	10.18 ± 0.53^∗^
MH+aspirin	9.14 ± 0.23^△^	9.65 ± 0.27^△^

Note: ^∗^*P* < 0.05 compared with the sham group at the same time point. ^△^*P* < 0.05 compared with the MH model group at the same time point.

**Table 4:** Measurement of CVF-NV (%) and CVF-T (%) in each group (mean ± SD, *n* = 9).

Groups	CVF-NV (%)		CVF-T (%)	
	4-week	8-week	4-week	8-week
Sham	3.35 ± 0.83	3.43 ± 0.86	7.95 ± 1.16	9.76 ± 1.75
MH model	7.16 ± 2.06^∗^	12.85 ± 2.03^∗^	27.31 ± 4.02^∗^	32.05 ± 6.86^∗^
MH+aspirin	4.64 ± 2.17^△^	4.70 ± 1.34^#^	8.46 ± 1.12^#^	11.55 ± 1.24^△^

Note: CVF: collagen volume fraction. CVF-NV: visual field collagen volume fraction percentage; CVF-T: total left ventricular myocardial collagen volume fraction. ^∗^*P* < 0.01 compared with the sham group at the same time point. ^△^*P* < 0.05 compared with the MH model group at the same time point. ^#^*P* < 0.01 compared with the MH model group at the same time point.

**Table 5:** Measurement of RVF-T (%) in each group (mean ± SD, *n* = 9).

Groups	RVF-T (%)	
	4-week	8-week
Sham	18.46 ± 1.85	19.30 ± 1.75
MH model	12.99 ± 3.72^∗^	12.06 ± 4.02^∗^
MH+aspirin	16.34 ± 1.13^△^	17.23 ± 1.12^#^

Note: RVF-T: volume percentage of reticular fibers. ^∗^*P* < 0.01 compared with the sham group at the same time point. ^△^*P* < 0.05 compared with the MH model group at the same time point. ^#^*P* < 0.01 compared with the MH model group at the same time point.

**Table 6:** Comparison of myocardial cell apoptosis rates (%) in each group (mean ± SD, *n* = 9).

Groups	4-week (%)	8-week (%)
Sham	5.06 ± 0.54	7.14 ± 0.71
MH model	12.52 ± 0.77^∗^	21.35 ± 0.86^∗^
MH+aspirin	8.93 ± 0.56^△^	11.64 ± 0.79^△^

Note: ^∗^*P* < 0.05 compared with the sham group at the same time point. ^△^*P* < 0.05 compared with the MH model group at the same time point.

**Table 7:** Changes of TNF-*α* in the serum and myocardial tissue in each group (mean ± SD, *n* = 9 (ng/L)).

Groups	Serum		Myocardial tissue	
	4-week	8-week	4-week	8-week
Sham	190.68 ± 59.15	210.86 ± 46.38	612.85 ± 94.63	658.82 ± 192.55
MH model	195.92 ± 42.68	212.49 ± 65.91	886.52 ± 246.59^∗^	972.89 ± 265.93^∗^
MH+aspirin	193.34 ± 56.05	236.07 ± 70.96	693.56 ± 172.08^△^	674.38 ± 186.26^△^

Note: ^∗^*P* < 0.05 compared with the sham group at the same time point. ^△^*P* < 0.05 compared with the MH model group at the same time point.

**Table 8:** Changes of IL-10 in the serum and myocardial tissue in each group (mean ± SD, *n* = 9 (ng/L)).

Groups	Serum		Myocardial tissue	
	4-week	8-week	4-week	8-week
Sham	58.91 ± 12.31	42.35 ± 15.05	245.75 ± 67.12	248.17 ± 56.58
MH model	64.18 ± 16.97	43.13 ± 13.72	246.48 ± 83.46	256.71 ± 48.97
MH+aspirin	61.78 ± 12.16	48.36 ± 12.26	250.13 ± 59.46	253.07 ± 38.09

**Table 9:** Changes of PGI_2_ in the serum and myocardial tissue in each group (mean ± SD, *n* = 9 (ng/L)).

Groups	Serum		Myocardial tissue	
	4-week	8-week	4-week	8-week
Sham	78.46 ± 23.92	86.18 ± 21.63	52.82 ± 23.75	172.71 ± 9.14
MH model	82.12 ± 20.01	69.54 ± 22.02^∗^	56.02 ± 26.37	147.69 ± 9.27^∗^
MH+aspirin	75.19 ± 24.48	89.97 ± 21.04^△^	49.86 ± 22.48	179.53 ± 7.29^△^

Note: ^∗^*P* < 0.05 compared with the sham group at the same time point. ^△^*P* < 0.05 compared with the MH model group at the same time point.

**Table 10:** Changes of TXA_2_ in the serum and myocardial tissue in each group (mean ± SD, *n* = 9 (ng/L)).

Groups	Serum		Myocardial tissue	
	4-week	8-week	4-week	8-week
Sham	328.59 ± 105.09	315.73 ± 98.13	1046.08 ± 158.60	962.74 ± 130.12
MH model	381.14 ± 112.10^∗^	434.31 ± 91.47^∗^	1168.21 ± 263.42^∗^	1250.49 ± 247.55^∗^
MH+aspirin	337.76 ± 116.07^△^	306.82 ± 110.07^△^	952.86 ± 207.35^△^	979.02 ± 142.25^△^

Note: ^∗^*P* < 0.05 compared with the sham group at the same time point. ^△^*P* < 0.05 compared with the MH model group at the same time point.

## Data Availability

The data that support the findings of this study are available on request from the corresponding author.
